# I can see clearly now: the effects of age and perceptual load on inattentional blindness

**DOI:** 10.3389/fnhum.2014.00229

**Published:** 2014-04-23

**Authors:** Anna Remington, Ula Cartwright-Finch, Nilli Lavie

**Affiliations:** ^1^Department of Psychology and Human Development, Centre for Research in Autism and Education (CRAE), Institute of Education, University of LondonLondon, UK; ^2^Institute of Cognitive Neuroscience, University College LondonLondon, UK

**Keywords:** perceptual load, inattentional blindness, development, attention, awareness, distractor, conscious perception

## Abstract

Attention and awareness are known to be linked (e.g., see Lavie et al., 2014, for a review). However the extent to which this link changes over development is not fully understood. Most research concerning the development of attention has investigated the effects of attention on distraction, visual search and spatial orienting, typically using reaction time measures which cannot directly support conclusions about conscious awareness. Here we used Lavie’s Load Theory of Attention and Cognitive Control to examine the development of attention effects on awareness. According to Load Theory, awareness levels are determined by the availability of attentional capacity. We hypothesized that attentional capacity develops with age, and consequently that awareness rates should increase with development due to the enhanced capacity. Thus we predicted that greater rates of inattentional blindness (IB) would be found at a younger age, and that lower levels of load will be sufficient to exhaust capacity and cause IB in children but not adults. We tested this hypothesis using an IB paradigm with adults and children aged 7–8, 9–10, 11–12 and 13 years old. Participants performed a line-length judgment task (indicating which arm of a cross is longer) and on the last trial were asked to report whether they noticed an unexpected task-irrelevant stimulus (a small square) in the display. Perceptual load was varied by changing the line-length difference (with a smaller difference in the conditions of higher load). The results supported our hypothesis: levels of awareness increased with age, and a moderate increase in the perceptual load of the task led to greater IB for children but not adults. These results extended across both peripheral and central presentations of the task stimuli. Overall, these findings establish the development of capacity for awareness and demonstrate the critical role of the perceptual load in the attended task.

## Introduction

Attention and awareness, though two distinct concepts, are intrinsically linked. The way in which they interact has been the subject of fierce debate over the past decades (Lavie et al., 2014). Anecdotally we know all too well that in some situations people appear to be completely unaware of anything outside their focus of attention (try talking to a child engaged in their favorite pursuit, for example playing their newest video game) yet in other situations people are constantly distracted and fail to focus. Even less is known about how the interplay between these processes is affected by development. The bulk of the previous research has assessed the development of attention using reaction time measures to examine age-related changes in susceptibility to distraction, visual search ability and spatial orienting. This research clearly demonstrates that control over selective attention and resistance from distraction develop with age (Plude et al., 1994). However it does not link attention to awareness as we briefly review below.

Studies of spatial attention and orienting demonstrate that while there is some evidence of mature spatial cueing effects from an early age (e.g., Brodeur and Enns, 1997), the reaction time costs associated with invalid cues appear to be far greater for children than adults (e.g., Pearson and Lane, 1990; Brodeur and Boden, 2000). This greater cost is thought to reflect children’s inability to disengage attention from the invalidly cued location and then redirect attention to the appropriate location. Moreover, children also fail to modulate their orienting responses in the face of varying cue predictability, suggesting that the control processes that govern orienting develop over childhood (Brodeur and Boden, 2000). However, the reaction time measures used in these studies do not tell us about the extent to which development of control over orienting attention leads to improved visual awareness.

Similarly, differences between adults and children are seen in Garner interference effects. Garner interference refers to the slowing of target responses caused by variation within an irrelevant dimension. For example, when shown cards that differ in color and value, the time taken to sort them on the basis of a relevant dimension (e.g., color) is greater when the irrelevant dimension (value) also differs from card to card (and *vice versa*) (Garner and Felfoldy, 1970). Such interference effects have been shown to vary developmentally: effects on response latencies were relatively large for younger children aged 4–5, 6–7 and 10–11 years (compared with adults), but gradually decreased with increasing age (e.g., Shepp and Swartz, 1976; Barrett and Shepp, 1988; Shepp and Barrett, 1991). This finding was particularly apparent when target and distractor dimensions were conjoined in one stimulus, and were therefore not easily separable. Together, these studies imply that the efficiency of gating irrelevant information is generally poorer at a young age but improves gradually with maturation. In addition, Stroop studies indicate that younger children are susceptible to greater interference than older children and adults, and this effect has been seen in both visual and auditory modalities (e.g., Hanauer and Brooks, 2003; and see MacLeod, 1991 for a review; Stroop, 1935; Posnansky and Rayner, 1977). Comalli et al. (1962) demonstrated that interference effects from incongruent words on color-naming latencies decreased with age throughout childhood and into adulthood (age range 7–80 years). Response competition studies also demonstrate that children (aged 4, 5 and 7 years) are less able than adults (aged 20 years) to filter out distractors (Enns and Akhtar, 1989) and that developmental trends in this ability are seen across childhood (5–12 years) (Enns and Girgus, 1985; Ridderinkhof et al., 1997).

While the evidence described so far demonstrates the development of attention control processes, it cannot inform us about the development of the capacity for visual awareness, because the aforementioned studies used indirect measures of perception (e.g., effects on target reaction times (RTs)) rather than direct measures (e.g., awareness reports). Effects on reaction times can be attributed to any processing component between the stimulus and response and clearly do not tell us about the extent of intrusions of the irrelevant stimuli into awareness. To the best of our knowledge there is only one study that has assessed relative awareness rates in children and adults. Memmert (2006) showed that, when asked to count ball passes between basketball players, children failed more often than teenagers (aged 13 years) and adults to notice a person dressed in a gorilla suit walking among the players (using the inattentional blindness (IB) video clip as used by Simons and Chabris, 1999). While this study only used one age group of children (8 years), and therefore developmental conclusions are limited, the finding that children had lower awareness reports than adults is potentially encouraging regarding the development of capacity for visual awareness.

Here we used Load Theory of Attention and Cognitive Control (Lavie et al., 2004) to address the development of the effects of attention on awareness within a framework that may able to provide a more comprehensive account for attention development than those just focused on attentional control. We suggest that cognitive maturation involves not only the development of attentional top-down control mechanisms that are responsible for preventing irrelevant distraction, but also the development of attentional capacity. Since according to Load Theory the level of perceptual processing that leads to awareness is determined by the availability of attentional capacity (e.g., Lavie et al., 2014), the developmental increase of this capacity should directly result in increased awareness rates with age. Note that for ease we shall use the contracted term “capacity for awareness” to refer to this linkage in the rest of the article. This has critical predictions for the development of attention and awareness as we outline below.

Load Theory states that focused selective attention (on task relevant rather than irrelevant information) depends not only on goal-directed cognitive control but also on the perceptual load (amount of potentially task-relevant information) of a given task. While full top-down cognitive control ability is necessary for the active maintenance of the current task priorities (including prioritization of relevant over irrelevant stimuli), this alone is insufficient to achieve exclusive focus on relevant items. In tasks of low perceptual load, spare capacity left over from the processing of task-relevant stimuli will “spill over” resulting in the perception of distractor stimuli. It is only when the perceptual load of the task is high enough to exhaust perceptual capacity that distractor perception—and their intrusions into awareness—will be prevented (Cartwright-Finch and Lavie, 2007; Macdonald and Lavie, 2008, 2011; Lavie et al., 2009; Carmel et al., 2011).

What are the implications for development? If attentional capacity for perception and awareness develops with age then we would expect first, that children will have lower levels of awareness than adults overall, and second, that a smaller increase in load will have a greater impact on reducing levels of awareness in children but not adults. These smaller increases in load would be sufficient to exhaust capacity in children, but not for adults who possess a larger capacity. At such levels adults are therefore expected to be more prone to the processing of irrelevant stimuli, whereas the children would be better able to focus and harder to detract from their task. Indeed, the studies showing children are more prone to distraction (Enns and Girgus, 1985; Enns and Akhtar, 1989; Ridderinkhof et al., 1997) involved a small number of stimuli in the display which would be expected to involve only a low level of load and therefore not to tax capacity.

Encouraging evidence for the suggestion that the capacity for perception increases with age has been obtained in a few previous studies. Multiple object tracking significantly increases from 7–22 years of age (Dye and Bavelier, 2010). Visual search literature indicates that adults perform visual search significantly better than children (e.g., Thompson and Massaro, 1989; Kaye and Ruskin, 1990; Brodeur et al., 1997) and that children show an increased search rate with age (Donnelly et al., 2007; Woods et al., 2013) and these age-related differences are particularly apparent in the more demanding search tasks—for example, in search for conjunction of features (color and orientation) rather than a single feature (color, orientation or size alone; Donnelly et al., 2007). The age-related improvement in visual search ability has been interpreted by some as evidence that selective attention and resistance to distraction develop with maturation. On closer consideration of the nature of visual search, however, it is clear that the non-target items in a visual search array are not irrelevant distractors, because they are task relevant (i.e., they have to be searched among in order to find the target, or to conclude that the target is absent from the array). Hence, these findings may indicate increased capacity for perceptual discrimination rather than improved ability to reject distractors. Moreover, as we discussed earlier, the reliance on RT measures in visual search studies falls short of providing direct evidence about perceptual processes related to conscious awareness.

Another line of support for our suggestion that attentional capacity for perception develops with ages comes from an event related potentials (ERP) study (Couperus, 2011) on the impact of perceptual load on neural markers of unattended perception in groups of children (7–18 years) and adults (mean age 24 years). Participants were asked to identify whether a character in the center of the screen was a letter or number, and load was manipulated by changing the stimulus duration (shorter presentation time representing higher load). Neural activity to an irrelevant character (a % sign presented offset from the central task) was recorded. Results demonstrated that for all age groups the amplitude of the P100 to unattended stimuli was lower under high load conditions than under low load conditions. However, for younger age groups the level of load needed to elicit these differences was far lower than for older children and adults.

Thus, overall we propose that development of selective attention involves both maturation of frontal cognitive control processes (as shown by the attention control RT studies) and an increase in perceptual capacity (as hinted by the motion tracking, visual search and recent ERP study). Interestingly, although maturation of the capacity for frontal cognitive control would allow older children to have better control over interference by irrelevant information that had been perceived, the development of perceptual capacity should lead to more cases of perception of irrelevant information, for example when the perceptual load of the task is sufficient to exhaust the smaller capacity of children and prevent awareness of additional stimuli, but leaves spare capacity in older children and adults who, consequently, can continue to process the irrelevant stimulus.

To date, only one study has begun to address this hypothesis. Huang-Pollock et al. (2002) tested children and adults on a visual search task with flanking distractors at varying levels of perceptual load. Their results showed that for all age groups, a distractor interference effect was seen at the lower levels of perceptual load and that this was eliminated at the highest level of load. The decline in interference effect, however, was seen at a lower level of load for the younger age groups. This is indicative of reduced capacity in these groups. As with many of the previous studies, however, the measures used by Huang-Pollock et al. were indirect (RT effects) and thus cannot support conclusions about awareness.

In the present study we therefore set out to test the effects of perceptual load on awareness using an IB paradigm (adapted from that used by Cartwright-Finch and Lavie, 2007) which directly assesses conscious awareness reports. Participants were asked to judge the line lengths of a cross shape with horizontal and vertical arms and an unexpected, irrelevant stimulus (a small gray square) was presented in the display on the final trial of the task. We assessed awareness for this irrelevant stimulus across a number of age groups at different levels of perceptual load. Perceptual load was varied by changing the relative lengths of the cross arms (more similar length in higher load conditions). Three levels of load were used: low and high load length parameters based on previous research (Cartwright-Finch and Lavie, 2007) and an intermediate load level (with length parameters in between those used for low and high based on pilot testing with children). Notice that the irrelevant stimulus used in this paradigm was not a strong competitor for attentional selection (e.g., it was not visually salient nor did it compete with the target response, c.f. Carmel et al., 2012). In this way our task allows us to clearly address perceptual capacity without the potential counter-effects of maturation of cognitive control functions (which are expected to improve distractor rejection). Importantly, any demands on cognitive control involved in the line-length discrimination task, did not vary across perceptual load conditions (since the task remained exactly the same, only the length of the lines varied). Thus effects of cognitive control could not confound or counter act the effects of perceptual load *per se*. If perceptual capacity does indeed increase with development then we expect the level of awareness for the irrelevant distractor to increase with age, and that a moderate increase in load will have a greater impact on the awareness rates at younger ages.

## Experiment 1

### Methods

In Experiment 1, levels of awareness were examined for an unexpected, peripheral square shape (critical stimulus) across various age groups under two different levels of perceptual load. Awareness for this critical stimulus (in this, and all experiments in this study) was assessed on the final trial as is customary in the IB paradigm (e.g., Mack and Rock, 1998). Note that the IB measure is solely based on that single report for each participant because once participants have been asked about their awareness one cannot be sure that on subsequent trials the stimulus will be entirely unattended to, hence the single critical-trial nature of the task.

#### Participants

Two-hundred and three participants were recruited from the Science Museum, London. After exclusions (see Results section), experimental age groups consisted of the following participants (*N*, mean age; SD): 7–8 year-olds (40, 7 years 11 m; 5.5 m), 9–10 year-olds (44, 9 years 11 m; 6.7 m), 11–12 year-olds (40, 12 years 0 m; 7.4 m), 13–14 year-olds (32, 14 years 0 m; 7.0 m), and adults (32, 30 years; 10 years). All participants reported normal or corrected-to-normal vision.

#### Stimuli and apparatus

**Figure 1:**
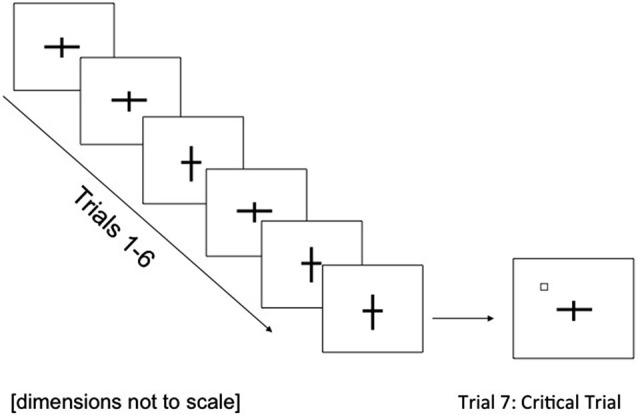
**Example of stimulus displays and trial sequence (low load) used in Experiment 1**. In the intermediate load condition, the cross arms were more similar in length (a difference of 1.9°).

The experiment was presented using E-Prime version 1.1 (Psychology Software Tools Inc.) on a PC connected to a 17″ monitor (1024 × 768 screen resolution; 75% contrast). Viewing distance was fixed at 60 cm with a chinrest. Stimulus displays were bitmap images created in Microsoft Paint and the background remained white throughout. Target displays consisted of a black cross at the center of the screen. One arm of the cross (either vertical or horizontal) subtended 3.9° whilst the shorter arm subtended either 0.7° (low load condition) or 2.0° (intermediate load condition). By using this intermediate level of perceptual load, the task is sensitive to developmental changes. Previous experiments (Lavie and Cox, 1997) have found no difference in the extent of distractor processing between low and intermediate levels of load (array sizes of 1, 2 and 4) in adults but differences have been seen in children (raising array size from 2 to 4 items; Huang-Pollock et al., 2002). On the seventh trial, a black outline square shape (sides subtending 0.3°) was presented in addition to the cross (see Figure 1). This critical stimulus appeared in one of four peripheral locations (counterbalanced between participants) all equidistant from fixation at 3.35° eccentricity, and positioned halfway between two neighboring cross-arms. A mesh pattern consisting of straight black lines of different orientations against the white background was used as a visual mask.

#### Procedure

Each trial proceeded as follows: small central fixation dot (1400 ms), blank interval (57 ms), fixation dot (97 ms), second blank interval (43 ms), centrally-located target cross (110 ms) and visual mask (496 ms). The repetition of the fixation dot and blank interval was in order that the fixation dot appeared to flicker, providing temporal warning for the onset of the task display. Participants were asked to indicate which arm of the cross was longer (horizontal or vertical) and responses were entered by the experimenter. All trials were initiated by the experimenter pressing the space bar. Participants were instructed to fixate centrally throughout and to guess if they were unsure.

Each participant completed 7 experimental trials: 6 non-critical trials and 1 critical trial. Within both non-critical and critical trials, the horizontal cross-arm was longer on half the trials (the vertical longer on the other half) with order counterbalanced across participants.

On the seventh trial, the critical stimulus was presented and the cross-task response was made and entered by the experimenter as normal. Immediately following response entry, participants were asked whether they noticed anything else appearing on the screen that had not been there before. Participants responded verbally giving details of the object if they could. The critical trial was then repeated in a final control trial. Before this trial, participants were instructed to ignore the cross and instead, look for anything extra that appeared in the display. Awareness for the critical stimulus was measured immediately after trial-termination by direct verbal report as before. Only participants reporting awareness for the critical stimulus on these control trials were included in the analysis.

### Results

**Figure 2:**
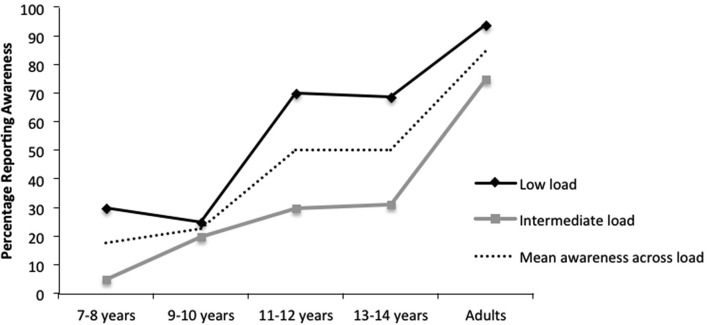
**Percentage of reported awareness of a peripheral critical stimulus (central target) as a function of perceptual load (low vs. intermediate) and age group, *N* = 188, in Experiment 1**.

Participants who failed the control trial (11), the critical trial target response (1), or to perform the task at all (1); and participants who gave uninterpretable responses (3) were excluded from the analyses. Remaining participants were divided among the experimental groups as follows: 7–8 years, low load (20) and intermediate load (20); 9–10 years, low load (24) and intermediate load (20); 11–12 years, low load (20) and intermediate load (20); 13–14 years, low load (16) and intermediate load (16); and adults, low load (16) and intermediate load (16). All participants included in the analyses performed the task adequately, with four or more correct line-length judgments entered. All of the participants who reported awareness of the critical stimulus (i.e., made a “Yes” response to the critical question) were able to describe correctly its location and at least two of its major features (shape, size or color). Figure 2 presents the percentage of reported awareness as a function of age (7–8 years, 9–10 years, 11–12 years, 13–14 years, adults) and perceptual load (low load, intermediate load). χ^2^-tests were performed on the data.

#### Overall rate of awareness

The analysis revealed a significant increase in overall rate of awareness reports with age, χ^2^ (4, *N* = 188) = 41.88, *p* < 0.001 (see Figure 2).

*Post-hoc* χ^2^ comparisons (with Bonferroni-Holm corrected α-levels for multiple comparisons) revealed similar levels of awareness at the two youngest age groups (7/40 reports in 7–8 year-olds vs. 10/44 reports in 9–10 year-olds, χ^2^ (1, *N* = 84) = 0.36, *p* = 0.55). However, children aged 9–10 years gave significantly lower proportions of aware reports than children aged 11–12 years (20/40 reports, χ^2^ (1, *N* = 84) = 6.79, *p* = 0.009). Children aged 11–12 years showed the same rates of awareness as children aged 13–14 years (16/32 reports, χ^2^ (1, *N* = 72) = 0, *p* = 1.00). However, children of 13–14 years gave significantly fewer reports of awareness than adults (27/32, χ^2^ (1, *N* = 64) = 8.58, *p* = 0.003). It seems, therefore, that capacity for awareness develops with age from 7 years old to adulthood.

This pattern was also seen at each load level: there was a significant increase in awareness reports with age under low load (χ^2^ (4, *N* = 96) = 26.42, *p* < 0.001) and intermediate load (χ^2^ (4, *N* = 92) = 22.16, *p* < 0.001). Under low load, specific comparisons showed significant increases from 9–10 to 11–12 year-olds (6/24 reports vs. 14/20 reports, χ^2^ (1, *N* = 44) = 8.91, *p* = 0.003) but no other significant differences between age groups (*p* > 0.60). Under intermediate load, a significant increase was seen between 13–14 year-olds and adults (5/16 reports vs. 12/16 reports, χ^2^ (1, *N* = 32) = 6.15, *p* = 0.013) with no other significant developmental changes (*p* > 0.10 in all other comparisons).

While this pattern of increase in awareness with age groups was expected with a moderate increase in level of load, it was somewhat unexpected that it was also found in the low load condition. However, note that despite the fairly evident difference in line length (difference of 3.2°) the low load task did take up some capacity. Moreover, recall that for the younger children we hypothesized that they have a smaller capacity. Therefore at a younger age, even low capacity consumption is likely to engage a larger proportion of their smaller total capacity, therefore having a larger effect on awareness for the unattended stimulus.

#### Impact of perceptual load

Significantly fewer participants reported awareness for the critical stimulus when performing a task of intermediate load (28/92) compared with a task of low load (52/96), χ^2^ (1, *N* = 188) = 10.82, *p* < 0.01. Importantly, comparing the effects of load for each age group revealed that increasing the perceptual load from low to intermediate level reduced awareness in all groups of children other than 9–10 year-olds: 7–8 years (6/20 vs. 1 /20 in low and intermediate load groups respectively, χ^2^ (1, *N* = 40) = 4.33, *p* = 0.037); 9–10 years (6 of 24 vs. 4 of 20, χ^2^ (1, *N* = 44) = 0.02, *p* = 0.69); 11–12 years (14/20 vs. 6 /20, χ^2^ (1, *N* = 40) = 6.40, *p* = 0.01); 13–14 years (11 of 16 vs. 5 of 16, χ^2^ (1, *N* = 32) = 4.5, *p* = 0.034). The lack of a significant load effect for participants aged 9–10 years is likely to be due to the low level of awareness under low load, producing a floor effect which limited any further reduction. As predicted, in the adult group, a moderate increase in the level of load did not affect awareness (15/16 vs. 12/16, χ^2^ (1, *N* = 32) = 2.1, *p* = 0.14) (see Figure 2).

A 2 × 5 multi-way frequency analysis of load (low, intermediate) by age (7–8, 9–10, 11–12, 13–14, adult) did not reveal a significant interaction, χ^2^ (4, *N* = 188) = 3.08, *p* = 0.54. However, inspection of Figure 2 suggests that this may be due to the restricted load effect caused by the low baseline level of awareness in the younger age groups. Indeed, when the two older age groups of children were combined (11–12 and 13–14) and compared with adults, the interaction of load (low, intermediate) by age (11–14, adults) reached significance, χ^2^ (1, *N* = 104) = 4.57, *p* = 0.027.

## Experiment 2

Experiment 1 demonstrates that awareness for stimuli outside the focus of attention develops with age, and moreover this is impacted by a small increase in the level of perceptual load suggesting decreased capacity for awareness in all children compared to adults. However, as the target was always presented in the center and the critical stimulus in the periphery, it is possible that there was a contribution to the overall effect of age on awareness from development of the ability to disengage from the focus on fixation (e.g., Pearson and Lane, 1990; Brodeur and Boden, 2000). To investigate this, in Experiment 2 we presented targets in the same range of peripheral positions as the critical stimulus in Experiment 1, to prevent engagement of a spatial focus that centered on fixation and excluded the periphery.

### Methods

#### Participants

Two-hundred and ten visitors to the Science Museum, London took part in this experiment. After exclusions (see Results section), participants (*N*, mean age; SD) were divided between the following age groups: 7–8 year-olds (40, 8 years 0 m; 6.7 m), 9–10 year-olds (44, 9 years 11 m; 7.0 m), 11–12 year-olds (40, 11 years 11 m; 7.1 m), 13–14 year-olds (36, 14 years 0 m; 7.0 m), and adults (32, 36 years; 11 years). All participants reported normal or corrected-to-normal vision.

#### Apparatus and procedure

Apparatus and procedure were as in Experiment 1.

#### Stimuli

**Figure 3:**
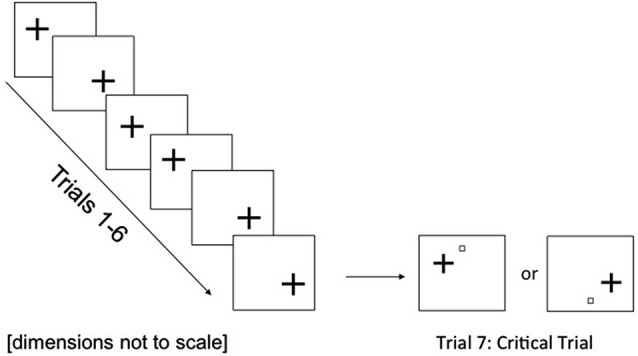
**Example of stimulus displays and trial sequence (intermediate load) used in Experiment 2**. On the critical trial, the cross was equally likely to appear in the top left or bottom right of the screen and the critical stimulus was always presented in the same quadrant as the cross.

Figure 3 presents an example of the stimuli used in this experiment. A fixation square subtending 1.4° was presented at the screen’s center. The size of the fixation square was chosen in order to eliminate the possibility of the initial fixation cue forward-masking critical stimuli that subsequently appeared at fixation (e.g., Breitmeyer, 1984). Target displays consisted of a black cross target with horizontal and vertical axes of the same size as those used in Experiment 1. Target crosses appeared in either one of two peripheral locations (upper-left or lower-right visual field, counterbalanced across trials) with the centers of each cross-target lying on an imaginary diagonal line, 3.35° away from fixation. In critical trials, a black outline square (each side subtending 0.3° as in Experiment 1) appeared in addition to the cross-target in one of two peripheral locations, 3.35° above or below fixation and 3.35° from center of target cross (position counterbalanced between participants, see Figure 3). All possible stimuli (peripheral cross-targets and peripheral critical stimuli) lay equidistant from one another. Thus, when the cross appeared in the lower-right position, peripheral critical stimuli were presented only in the lower visual field and *vice versa* for upper visual field stimuli. The visual mask from Experiment 1 was used. A white background was maintained throughout (see Figure 3).

Target position was counterbalanced across participants with targets appearing in the upper visual field position on half the trials and in the lower position on the other half of trials. Target crosses were presented in the same position on the sixth and seventh (critical) trials for one group of participants (e.g., upper position followed by upper position) and in different positions to another group (e.g., upper position followed by lower position).

### Results

Participants who failed the visual control trial (7), the main target task (2); the critical trial target response (2); participants who gave uninterpretable responses (2); participants who were not naive to the experiment (3); and participants who could not understand instructions (2) were excluded from the analyses. Remaining participants were as follows: 7–8 years, low load (20) and intermediate load (20); 9–10 years, low load (24) and intermediate load (20); 11–12 years, low load (20) and intermediate load (20); 13–14 years, low load (16) and intermediate load (20); and adults, low load (16) and intermediate load (16).

#### Overall awareness

**Figure 4:**
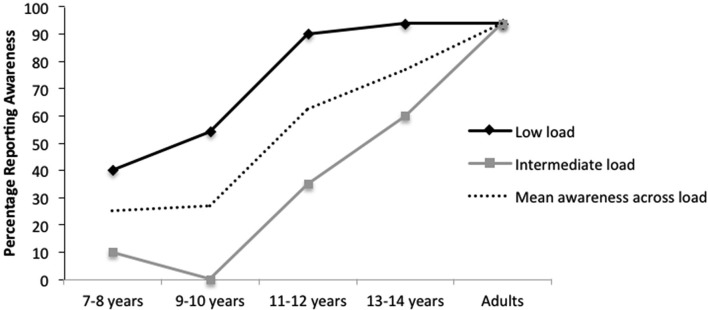
**Percentage of reported awareness of a peripheral critical stimulus (peripheral target) as a function of perceptual load (low vs. intermediate) and age, *N* = 192, in Experiment 2**.

Figure 4 presents the percentage of reported awareness for the critical stimulus as a function of age and perceptual load (low load vs. intermediate load). All of the participants who reported awareness of the critical stimulus (i.e., made a “Yes” response to the critical question) were able to describe correctly its location and at least two of its major features (shape, size or color).

χ^2^ analyses revealed that rates of awareness reports significantly increased with age across participants aged 7–8 years to adults, (χ^2^ (4, *N* = 192) = 52.13, *p* < 0.0001). The effect of age on awareness found in Experiment 1 is therefore replicated in the current experiment where both the attended cross-targets and the critical stimulus for which awareness was measured were presented in the periphery.

*Post-hoc* comparisons of awareness rates between different age groups (with Bonferroni-Holm corrected α-levels for multiple comparisons) revealed no difference in reported awareness between 7–8 year-olds (10/40) and 9–10 year-olds (13/44), (χ^2^ (1, *N* = 84) = 0.22, *p* = 0.64). However, rates of awareness reports increased significantly from children aged 9–10 years to 11–12 years (25/40, χ^2^ (1, *N* = 84) = 9.19, *p* = 0.002). Again, as in Experiment 1, there was no difference between the rates of awareness reports given by 11–12 year-olds and 13–14 year-olds (27/36, χ^2^ (1, *N* = 76) = 1.37, *p* = 0.24), and in this experiment children aged 13–14 years did not report awareness significantly less often than adults (30/32, χ^2^ (1, *N* = 68) = 4.39, *p* = 0.04). Therefore, in this experiment, rates of awareness rose with age between the age brackets of 7–10 years and 11–14 years (see Figure 4).

As in Experiment 1, significant developmental increases in awareness reports were evident under both low load (χ^2^ (4, *N* = 96) = 24.60, *p* < 0.001) and intermediate load (χ^2^ (4, *N* = 96) = 44.42, *p* < 0.001). Under low load, however, specific comparisons showed a significant increase only from 9–10 year-olds to 11–12 year-olds (13/24 reports vs. 18/20 reports, χ^2^ (1, *N* = 44) = 6.73, *p* = 0.009) with no other significant differences between age groups (*p* > 0.05). Conversely, under intermediate load, a significant increase was seen between 9–10 to 11–12 year-olds (0/20 reports vs. 7/20 reports, χ^2^ (1, *N* = 40) = 8.49, *p* = 0.003) and between 13–14 year-olds and adults (12/20 reports vs. 15/16 reports, χ^2^ (1, *N* = 36) = 5.40, *p* = 0.002). Thus development of awareness outside the focus of attention is also evident in tasks that do not require disengagement from fixation.

#### Impact of perceptual load

The χ^2^ analysis also revealed that significantly fewer awareness reports were given in the intermediate load group (36/96) than the low load group (69/96), χ^2^ (1, *N* = 192) = 22.89, *p* < 0.001. These results replicate the previous findings regarding the effects of load on awareness in Experiment 1.

Separate χ^2^ analyses showed that increasing perceptual load from low level to an intermediate level in the cross task caused a significant reduction in awareness for the critical stimulus at every age group of children. Awareness reports decreased from low load (8/20) to intermediate load (2/20) for 7–8 year-olds, (χ^2^ (1, *N* = 40) = 4.8, *p* = 0.03); 9–10 year-olds, (13/24 vs. 0/20, χ^2^ (1, *N* = 44) = 15.38, *p* < 0.001); 11–12 year-olds (18/20 vs. 7/20, χ^2^ (1, *N* = 40) = 12.91, *p* < 0.001); and 13–14 year-olds (15/16 vs. 12/20, χ^2^ (1, *N* = 36) = 5.4, *p* = 0.02). By contrast, there was no difference in the rates of awareness reported by adults under conditions of low load (15/16) vs. intermediate load (15/16) (see Figure 4). Thus children of ages up to 16 are more affected by an intermediate increase in the level of load, when compared to adults.

A 2 × 5 multi-way frequency analysis on the interaction of load (low, intermediate) by age (7–8, 9–10, 11–12, 13–14, adult) did not reach significance, χ^2^ (4, *N* = 192) = 7.70, *p* = 0.10. However, a multi-way frequency analysis of load (low, intermediate) by age (9–10, 11–12, 13–14, adult) excluding the 7–8 year age group (which showed smaller effects of load on awareness, likely to be due to low baseline awareness levels) revealed a significant interaction, χ^2^ (3, *N* = 152) = 8.25, *p* = 0.04. This interaction illustrated in Figure 4 suggests that the effect of load on awareness became smaller as age increased.

The convergence of results across both tasks that involve and do not involve disengagement from fixation suggests an overall reduction in awareness outside the focus of attention that does not depend on the development of the ability to disengage from fixation. Overall, these findings support our hypothesis of smaller perceptual capacity in younger children compared to older children and in all children compared to adults.

## Experiment 3

Experiments 1 and 2 demonstrate that for adults a moderate increase in the level of load did not affect the level of awareness for an unexpected critical stimulus. Based on previous research with similar tasks (Lavie and Cox, 1997; Cartwright-Finch and Lavie, 2007) we predicted that it would take a higher level of perceptual load to reduce awareness in a group of adult participants. To test this, and to confirm that for the tasks and experimental conditions (e.g., testing in the museum) used in Experiments 1 and 2 load does indeed modulate awareness in adulthood, we carried out a further task on an adult group with a higher level of perceptual load.

### Methods

#### Participants

Sixty-nine visitors to the Science Museum, London participated in the experiment. All reported normal or corrected-to-normal vision and were between 18–47 years old.

#### Apparatus and procedure

Apparatus and procedure were as in Experiment 1.

#### Stimuli

Stimuli were as in Experiment 2, but with low load (longer arm of cross subtended 3.9°; shorter arm subtended 0.7°) and high load (longer arm of cross subtended 3.9°; shorter arm subtended 3.31°) trials.

### Results

All participants performed the task adequately, with four or more correct line-length judgments entered. Excluded were participants who failed the final control trial (2), participants who provided unclear responses (2), and one participant who did not understand the awareness questioning. Remaining participants were divided equally between the two experimental groups: low load (16) and high load (16). All of the participants who reported awareness of the critical stimulus (i.e., made a “Yes” response to the critical question) were able to describe correctly its location and at least two of its major features (shape, size or color). χ^2^ analyses revealed that rates of awareness reports were significantly higher in the low load condition (15/16) than in the high load condition (2/16), (χ^2^ (2, *N* = 32) = 21.20, *p* < 0.01).

**Figure 5:**
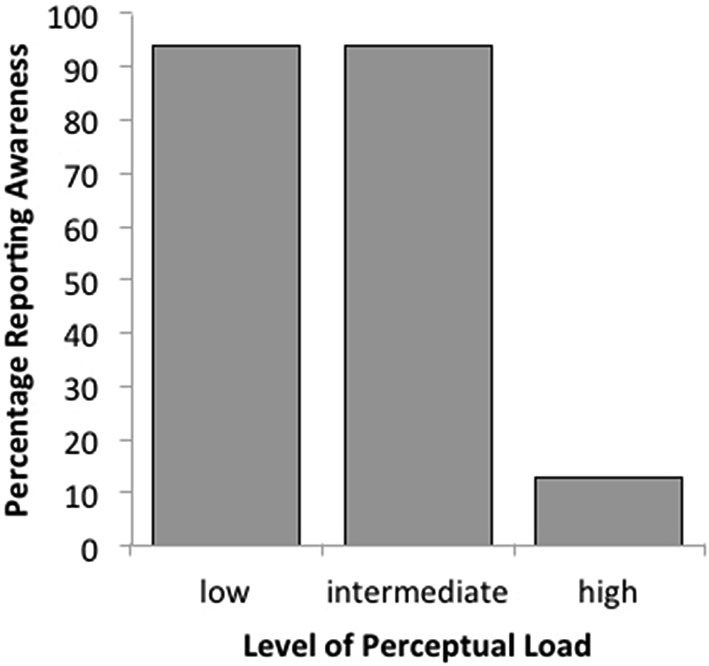
**Percentage of reported awareness of a peripheral critical stimulus (peripheral target) as a function of perceptual load (low vs. intermediate vs. high) in adults (Experiments 2 and 3)**.

Combining these data with those from the intermediate level of load in Experiment 2, one can see that rates of awareness under low (15/16) and intermediate (15/16) levels of load are equivalent and both are significantly greater than awareness in the high load condition (2/16) (see Figure 5). Experiment 3 confirms that increasing the level of load to a greater extent results in modulation of awareness for the critical stimuli in adult participants.

## Discussion

Our results reveal that awareness outside the focus of attention develops with age and that a small increase in the perceptual load of the attended task reduces awareness rates for children (aged 7–14 years) but not adults. The effects of age and load on awareness for a stimulus in the visual periphery converged across tasks that involved disengagement from fixation (Experiment 1) and tasks that did not involve this disengagement (Experiment 2). This demonstrates an overall increase in awareness outside the focus of attention with age. Note that this increase in awareness with age was found in tasks involving low and moderate levels of load. The fact that younger children were more prone to IB in such tasks provides support for our hypothesis that perceptual capacity is increased in older children compared to younger children with these levels of load disproportionately taxing the smaller capacity of the younger ages. This finding—combined with the fact that, across development, a small increase in the level of load that had no effect on awareness in adults significantly reduced the awareness for an unexpected critical stimulus—demonstrates a smaller perceptual capacity in younger children compared to older children and in all children compared to adults. In adults however, a higher level of perceptual load was required to modulate awareness (Experiment 3). This finding supports our proposal that development of attention involves maturation of awareness for information that is not part of the attended task. Consequently, the development of perceptual capacity plays an important part in the development of attention, in addition to the well-known development of cognitive control. An increase in capacity would lead to greater availability of resources, resulting in less of an impact (proportionately) of increases in the level of load.

It is perhaps important to note that alternative accounts for the present findings in terms of inability to follow verbal probing and instruction at younger ages are highly unlikely for the following reasons. All participating children were school age, which in the UK means they already have had 3–6 years of formal education (performance of 7–8 and 9–10 year-olds did not differ) and therefore much experience following verbal instructions. Moreover, due to exclusion criteria, all these children performed correctly on the control trial (and the cross task generally), indeed only 11 of 203 (Experiment 1) and 7 of 210 children (Experiment 2) were excluded for failing the control trial (failures that may have resulted from undiagnosed reduced visual acuity). Furthermore, the experimenter’s notes indicated that the children of all ages generally expressed a high level of confidence in their awareness reports: typically either a clear “yes” and a point to the area in the screen where the critical stimulus appeared, or a flat “no”. This suggests that they have clearly understood the question, and perhaps also that responses tended to be a direct reflection of their perceptual trace rather than the result of elaborate deliberation. This goes some way towards alleviating concerns regarding response criteria, although of course without formally measuring sensitivity verses criterion (e.g., using signal detection paradigms) alternative accounts for awareness in terms of effects on response criteria remain viable. An important extension of this work would be to examine the effects of load and age on awareness reports using a signal detection paradigm.

The development of global vs. local processing should also be considered here. Previous research has highlighted age-related changes in the bias for processing local over global scene features (and *vice versa*). Predominantly, a local processing bias is noted early in development with a global precedence effect emerging from age 5 (e.g., Poirel et al., 2008; Vinter et al., 2010), perhaps a consequence of immature hemispheric communication at younger ages (Moses et al., 2002; and see Smith and Chatterjee, 2008, for a review). Moreover, some suggest that this global precedence can in fact only be seen much later in development, with local precedence appearing to dominate perceptual organization until adolescence, followed by a gradual transition to more a global processing style (Dukette and Stiles, 1996; Mottron et al., 1999, 2003; Enns et al., 2000; Porporino et al., 2004; Kimchi et al., 2005; Scherf et al., 2009). However, evidence for the reverse pattern has also been found, with demonstrations that 6 and 10 year-olds showed strong global bias, stronger even than that of adults (Mondloch et al., 2003), and that infants show greater sensitivity to global than to local structure in visual stimuli (Quinn and Eimas, 1986; Ghim and Eimas, 1988; Freeseman et al., 1993; Quinn et al., 1993; Frick et al., 2000). It has been argued that this contradiction may be due to differences in task parameters that differentially affect children’s perceptual biases (Scherf et al., 2009).

Could these developmental changes in hierarchical processing account for the better ability to detect the critical stimulus in the periphery at older ages? For example, could the development of awareness of the critical stimulus in the periphery be attributed to the development of a more global processing strategy that encompasses both the task stimuli at fixation and the periphery? This is somewhat unlikely for the following reasons. The critical stimulus was in fact smaller in size than the lines used in the line-length discrimination task. It is therefore unclear whether development of global precedence with age would lead to increased awareness of this stimulus. In addition, the convergence of results across Experiment 2 (which, notwithstanding our first point, could be said to encourage a more global processing strategy because of the reduced spatial certainty and the presence of all stimuli in the periphery) and Experiment 1 (which could be said to encourage a more spatially-local processing strategy) suggest that this factor was unlikely to play a crucial role. Nevertheless, future research assessing awareness in tasks that involve both local and global elements of task displays under various levels of load (e.g., Navon Figures) could prove interesting in revealing any potential interactions between these factors.

### Increased focus in younger children

It is interesting to consider the flipside of the capacity development established in this study. The age-related increases in awareness outside the focus of attention demonstrate that there will be more cases where younger children are unaware of stimuli outside their more narrow focus of attention. In such situations younger children will appear to have a higher level of focus than older children, and under moderate increases in the task load children will be more focused than adults. This fits with anecdotal observations that children can appear more oblivious to their surroundings, and be harder to detract from their current object of focus.

Indeed, our conclusions are consistent with Memmert’s (2006) findings (discussed earlier) that 8 year-old children exhibited lower awareness rates than 13 year-olds and adults on the IB task with the gorilla. However, the dynamic video clip paradigm that was used in this study allowed participants to freely move their eyes. Thus any reports of blindness to the “gorilla” while tracking the ball passes may be due to blurring on the retina caused by intersaccadic suppression during eye movement rather than inattention. In keeping with the well-established finding that orienting of gaze matures with age (Pearson and Lane, 1990), it is likely that children’s reduced ability to reorient gaze from the ball to the “gorilla” has contributed to the observed differences in awareness between the age groups. In contrast, our task used short exposure durations, which preclude eye movement, and thus ensure that our findings reflect attentional effects rather than eye movements.

Our findings can therefore be more clearly attributed to a narrowing of attention which leads to the exclusion of irrelevant information under higher levels of perceptual load. Importantly this effect requires only small increases in perceptual load for children.

Note that although rates of IB were increased overall for the younger children (including in the low load condition) this does not suggest the low level of load was sufficient to exhaust their smaller capacity since a further reduction in awareness was found with the moderate increase in load for all ages, including the youngest. Indeed, an interesting prediction arises whereby finer grained increases in the levels of perceptual load would be sufficient to exhaust capacity for children of younger, but not older, ages. This would be an interesting direction for future research.

### Reconciling increased focus and reduced cognitive control

How do we reconcile the fact that children have, in some situations, higher levels of focus and yet reduced cognitive control? The key is firstly to consider the nature of the irrelevant distractor item. Cognitive control processes are only required to control against distractors that compete with the target stimulus for selection—for example, when the distractor is associated with a different target response or is a very salient stimulus such as a face. However, non-competing stimuli do not require cognitive control over selection. Therefore, when cognitive control processes are loaded (e.g., working memory) there is an increase in the level of processing of items that compete for the response, but not of non-competing irrelevant information (Carmel et al., 2012). This finding, together with our application of load theory to development suggests that smaller increases in cognitive control load will lead to increased distraction and greater processing of irrelevant competing distractors by younger children due to cognitive control immaturity.

In our study, however, the irrelevant distractor is non-competing as it is a small square presented while participants are performing a line discrimination task. The processing of non-competing distractors does not depend on cognitive control but depends instead on perceptual processes. With this in mind, a different developmental pattern of results is expected for the processing of these stimuli. Under low load (i.e., when perceptual capacity limits are not reached), we would not expect to see increased processing of irrelevant non-competing items with maturation. Indeed, we would expect this equivalent performance across age groups to remain even if cognitive control processes were loaded. Comparing our findings under low load with those of Huang-Pollock et al. (2002) demonstrates this dichotomy. In their study, the distractor competed for a response (letter distractor and letter search attended task) and, as predicted, under low load the children showed increased distractor processing. This interference was then eliminated by a smaller increase in perceptual load (than for adults). In our study, however, the irrelevant item was non-competing and we show the opposite pattern—distractor processing was lower in the children than the adults under both low and intermediate levels of perceptual load. While this was expected under intermediate levels of load (where the task loaded the children’s smaller capacity, but not that of the adults) it was not predicted that age-related changes would be evident under low load. As mentioned above, the lack of equivalent performance in this condition is likely due to age-related changes in the ability to disengage from the cross stimulus and shift the focus of attention. Future research involving the manipulation of cognitive control vs. perceptual load, and competing vs. non-competing distractors, would undoubtedly be fruitful.

### Implications

The results we present here have implications for attentional performance in a number of situations. Given that a moderate rise in perceptual load leads to a greater increase in focused attention for younger children (compared to older children and adults), it is likely that performance on paradigms such as Garner and Stroop tasks could be improved in younger age groups by the addition of perceptual load—provided that these are performed with some separation between the relevant and irrelevant dimensions so that attention can be clearly engaged in the relevant task rather than the irrelevant processing with increased perceptual load. Aside from these experimental situations, our findings also have practical consequences whereby the modulation of perceptual load could be used to improve the focus of attention in young children, and hence the efficacy of learning tools.

The present findings that children have lower rates of awareness than adults and that a moderate increase in perceptual load that had no effect on awareness in adults, was sufficient to increase IB rates in children also have interesting neural implications. They imply that unattended stimuli in IB tasks should evoke less activity in children than adults due to their smaller perceptual capacity. Furthermore, these neural modulations would be found at lower levels of load in children, compared to the level needed to reduce neural response to unattended stimuli in adults.

Overall, our findings demonstrate that maturation involves the development of awareness outside the focus of attention and an increase in perceptual capacity. This leads to a greater likelihood of IB, namely enhanced focus and resistance to irrelevant intrusions into awareness in the younger children. This must be considered together with evidence of age-related changes in cognitive control processes to understand fully the development of selective attention. Looking at the development of the latter in isolation does not accurately reflect the full picture.

## Conflict of interest statement

The authors declare that the research was conducted in the absence of any commercial or financial relationships that could be construed as a potential conflict of interest.
